# Supplementary material for: Nicotinamide Mononucleotide Prevents Free Fatty Acid-Induced Reduction in Glucose Tolerance by Decreasing Insulin Clearance
Source: Int J Mol Sci. 2021 Dec 8;22(24):13224. doi: 10.3390/ijms222413224 (PMC8709165; doi:10.3390/ijms222413224)
Supplement: Supplementary file 1 [file ijms-22-13224-s001.zip › ijms-1495151-supplementary.pptx]

## Slide 1
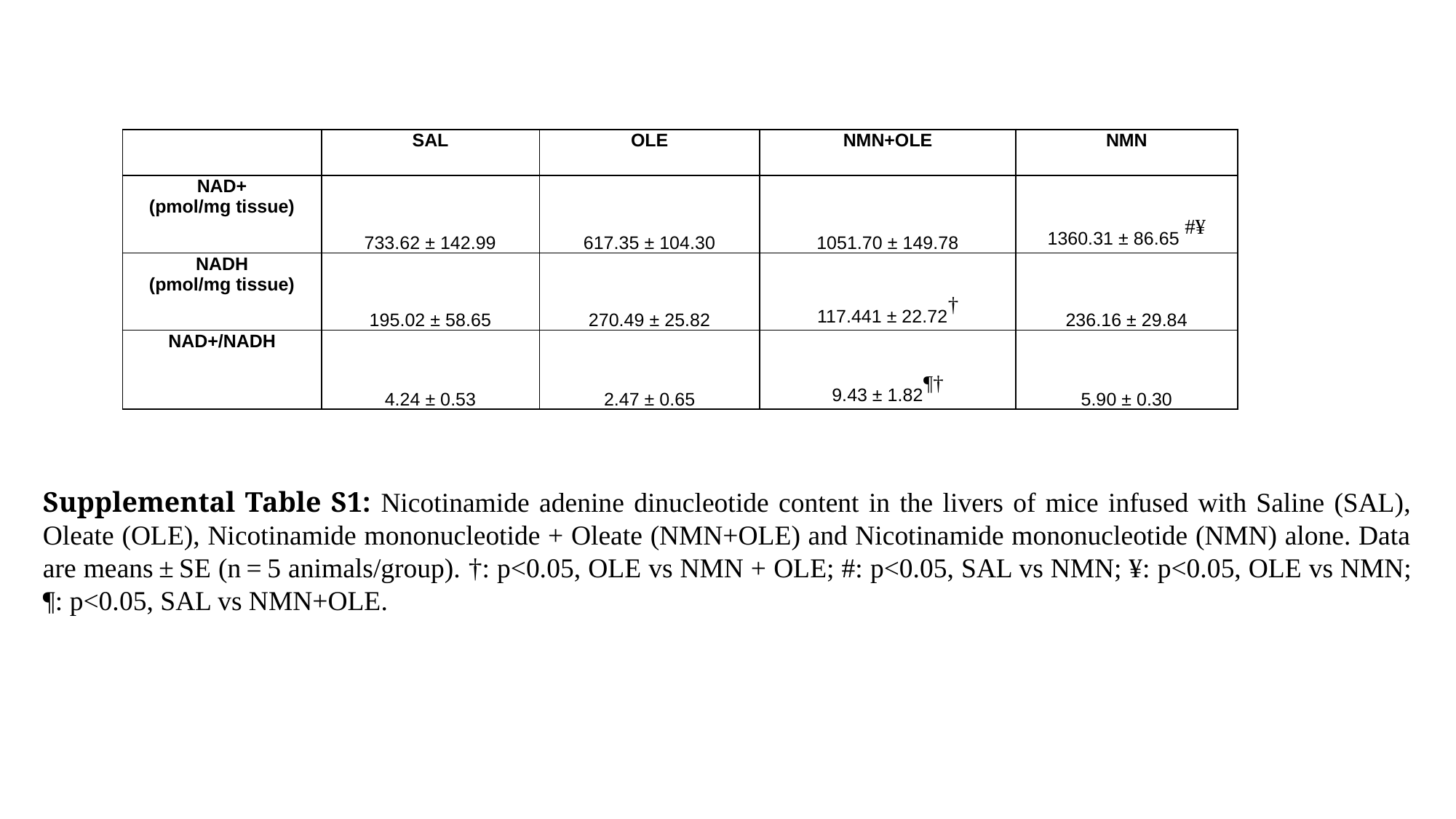

| | SAL | OLE | NMN+OLE | NMN |
| --- | --- | --- | --- | --- |
| NAD+ (pmol/mg tissue) | 733.62 ± 142.99 | 617.35 ± 104.30 | 1051.70 ± 149.78 | 1360.31 ± 86.65 #¥ |
| NADH (pmol/mg tissue) | 195.02 ± 58.65 | 270.49 ± 25.82 | 117.441 ± 22.72† | 236.16 ± 29.84 |
| NAD+/NADH | 4.24 ± 0.53 | 2.47 ± 0.65 | 9.43 ± 1.82¶† | 5.90 ± 0.30 |
Supplemental Table S1: Nicotinamide adenine dinucleotide content in the livers of mice infused with Saline (SAL), Oleate (OLE), Nicotinamide mononucleotide + Oleate (NMN+OLE) and Nicotinamide mononucleotide (NMN) alone. Data are means ± SE (n = 5 animals/group). †: p<0.05, OLE vs NMN + OLE; #: p<0.05, SAL vs NMN; ¥: p<0.05, OLE vs NMN; ¶: p<0.05, SAL vs NMN+OLE.

## Slide 2
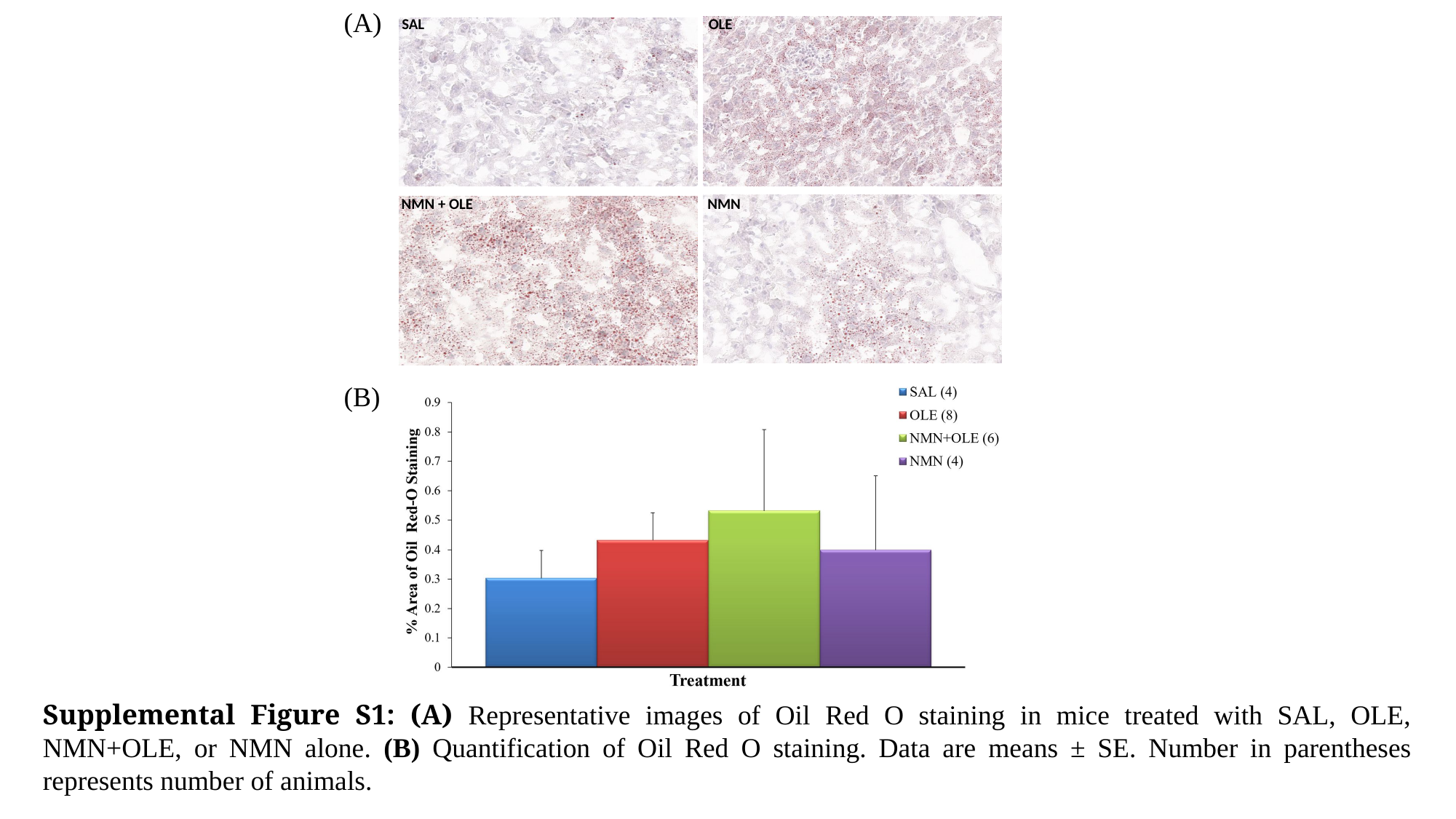

(A)
SAL
OLE
NMN + OLE
NMN
(B)
Supplemental Figure S1: (A) Representative images of Oil Red O staining in mice treated with SAL, OLE, NMN+OLE, or NMN alone. (B) Quantification of Oil Red O staining. Data are means ± SE. Number in parentheses represents number of animals.

## Slide 3
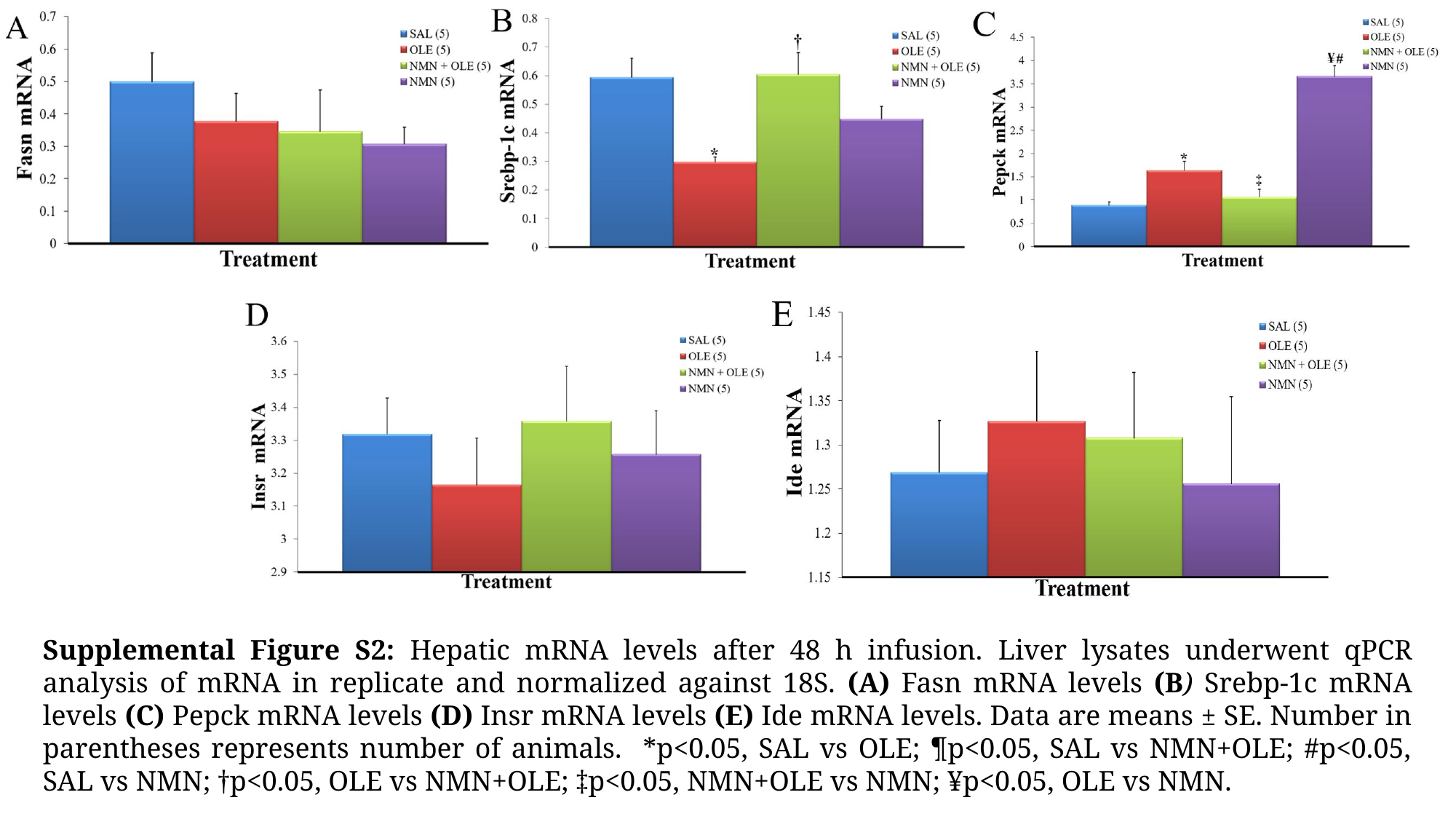

Supplemental Figure S2: Hepatic mRNA levels after 48 h infusion. Liver lysates underwent qPCR analysis of mRNA in replicate and normalized against 18S. (A) Fasn mRNA levels (B) Srebp-1c mRNA levels (C) Pepck mRNA levels (D) Insr mRNA levels (E) Ide mRNA levels. Data are means ± SE. Number in parentheses represents number of animals. *p<0.05, SAL vs OLE; ¶p<0.05, SAL vs NMN+OLE; #p<0.05, SAL vs NMN; †p<0.05, OLE vs NMN+OLE; ‡p<0.05, NMN+OLE vs NMN; ¥p<0.05, OLE vs NMN.

## Slide 4
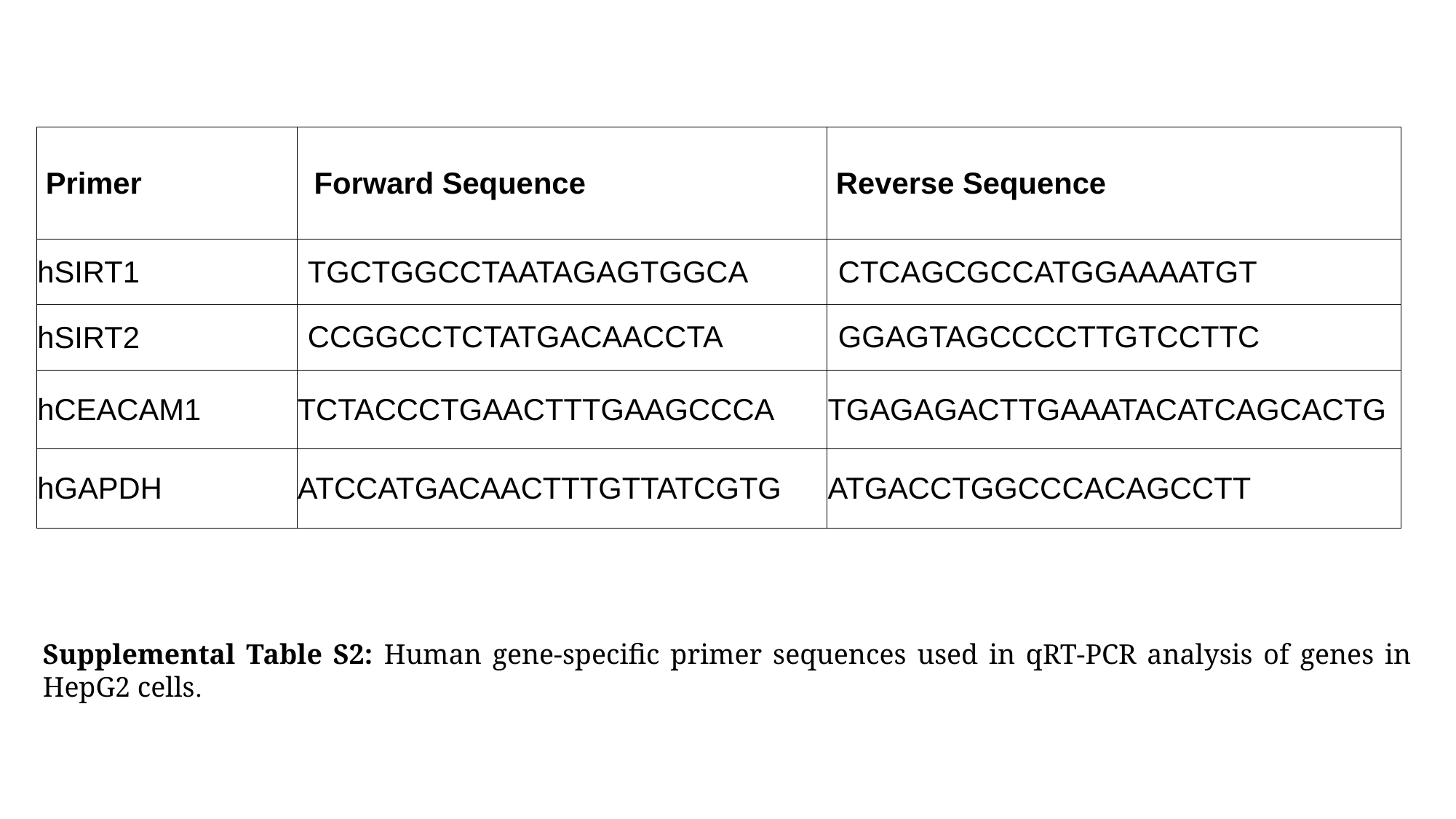

| Primer | Forward Sequence | Reverse Sequence |
| --- | --- | --- |
| hSIRT1 | TGCTGGCCTAATAGAGTGGCA | CTCAGCGCCATGGAAAATGT |
| hSIRT2 | CCGGCCTCTATGACAACCTA​ | GGAGTAGCCCCTTGTCCTTC​ |
| hCEACAM1 | TCTACCCTGAACTTTGAAGCCCA | TGAGAGACTTGAAATACATCAGCACTG |
| hGAPDH | ATCCATGACAACTTTGTTATCGTG | ATGACCTGGCCCACAGCCTT |
Supplemental Table S2: Human gene-specific primer sequences used in qRT-PCR analysis of genes in HepG2 cells.

## Slide 5
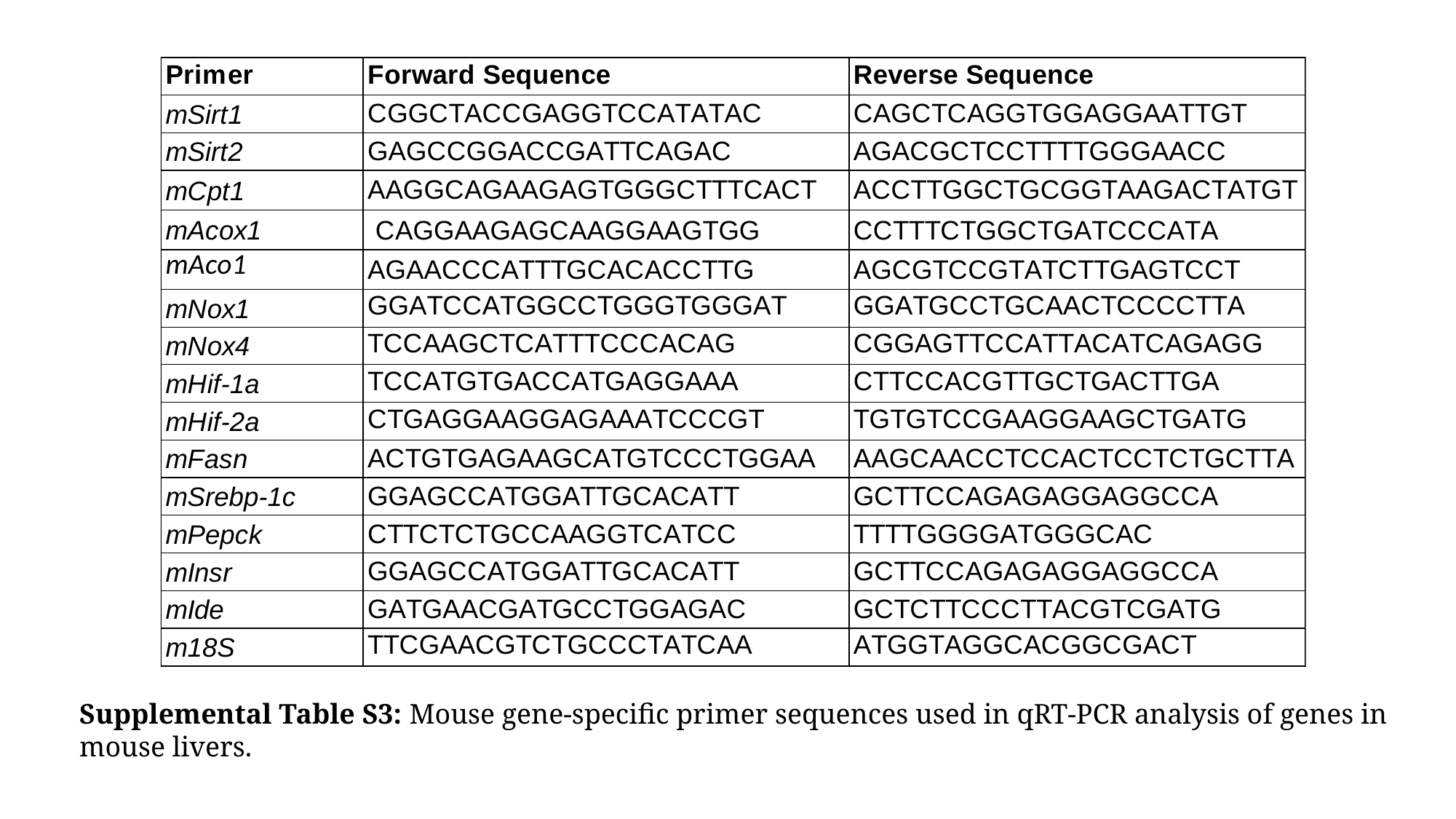

Supplemental Table S3: Mouse gene-specific primer sequences used in qRT-PCR analysis of genes in mouse livers.
